# Work-related stress in intensive care unit night shift nurses: A cross-sectional analysis of prevalence and determinants

**DOI:** 10.1371/journal.pone.0336041

**Published:** 2025-11-10

**Authors:** Fadia Ahmed Abdelkader Reshia, Asmaa Ibrahem Abo Seada, Basma Salameh, Jihad Abdallah, Eman Mayouf Ruqad Alruwaili, Ebtsam Salah Shalaby Salama, Wejdan Ghazi Alrumieh, Asmaa Mohamed Ahmed Elnosary

**Affiliations:** 1 Medical Surgical Nursing, College of Nursing, Jouf University, Sakaka, Saudi Arabia; 2 Critical Care and Emergency Nursing, Faculty of Nursing, Mansoura University, Mansoura, Egypt; 3 Professor of Nursing/Critical Care Nursing, Faculty of Nursing, Arab American University, Jenin, Palestine; 4 Department of Agricultural Engineering, Animal Genetics and Biostatistics, An-Najah National University, Nablus, Palestine; 5 Nursing Specialist, Cardiac Center, King Abdulaziz Specialist Hospital, Sakaka, Saudi Arabia; 6 Psychiatric and Mental Health Nursing, Faculty of Nursing, Mansoura University, Mansoura, Egypt; 7 Head Nurse Pediatric Intensive Care Unit, Women, Maternity and Children’s Hospital, Sakaka, Saudi Arabia; 8 Critical Care and Emergency Nursing, Faculty of Nursing, Mansoura University, Mansoura, Egypt; Ajman University, UNITED ARAB EMIRATES

## Abstract

**Background:**

Night-shift ICU nurses are highly vulnerable to work-related stress, impacting performance, patient safety, and burnout risk.

**Aim:**

To assess the prevalence and sources of work-related stress among ICU nurses working night shifts and to explore the relationships between stress levels and selected demographic and professional variables.

**Method:**

A descriptive cross-sectional study was conducted with a convenience sample of 362 ICU nurses working at Mansoura University Hospitals. Participants completed a two-part questionnaire comprising a nurses’ demographic data sheet and a validated Arabic version of the Revised Nursing Stress Scale (RNSS). Data were collected via a Google Form between January 10, 2025, and April 30, 2025, and analyzed using SPSS version 21.0, employing descriptive statistics, t-tests, ANOVA, and reliability testing.

**Results:**

Nearly 60% of participants reported moderate to high levels of stress. The highest stress scores were associated with workload, interactions with patients and their families, and supervisory issues. Female nurses reported significantly higher stress levels related to death and dying, patient-family interactions, and discrimination (p < 0.05). Nurses with less than five years of experience reported significantly greater stress concerning uncertainty about treatment. No significant associations were found between overall stress levels and variables such as age, marital status, educational level, or frequency of night shifts.

**Conclusion:**

Work-related stress is highly prevalent among ICU nurses working night shifts, primarily driven by heavy workloads and interpersonal challenges. Stress levels vary significantly by gender and years of experience, underscoring the need for organizational interventions, such as staff support programs, training, and effective shift management, to mitigate stress and improve the quality of care. Future research is warranted to conduct longitudinal assessments of stress and to evaluate the effectiveness of interventions across diverse healthcare settings.

## Background

The Intensive Care Unit (ICU) is a highly demanding environment where nurses care for critically ill patients under intense physical and emotional pressures [1,2]. Night shift ICU nurses face additional challenges, including disrupted circadian rhythms, fatigue, and extended working hours, which heighten vulnerability to work-related stress [3–5]. This stress impairs cognition (attention, memory, decision making), and is linked to reduced clinical performance, medication/medical errors, and higher near-miss incidents experienced by highly stressed nurses [5–9]. Occupational stress is the chronic strain that arises when workplace demands exceed coping capacity, with adverse effects on well-being, productivity, and organizational efficiency [10]. Major contributors in ICUs include high responsibility, emotional burden, poor interprofessional communication, limited support, exposure to unexpected deaths or painful procedures, and complex interactions with family members, compounded by inadequate work environments [11,12].

ICU nurses play a vital role in providing continuous care within the healthcare system [13]. Those working night shifts are particularly vulnerable to stress due to high patient acuity, excessive workload, and the need to maintain concentration during prolonged 12-hour shifts [14]. Night work disrupts sleep patterns and circadian rhythms, negatively affecting both physical and psychological health [4]. The ICU environment further intensifies these challenges, as life-threatening cases demand constant vigilance and rapid decision-making [15]. Prolonged exposure to such stressors contributes to exhaustion, depersonalization, and emotional strain, ultimately resulting in burnout, characterized by emotional exhaustion and a decline in personal accomplishment [3,15,16].

Extended 12-hour shifts and limited control over scheduling heightened stress and burnout, while scheduling flexibility can mitigate, though not eliminate, these effects [17]. Perceived organizational support (rest breaks, peer support, and access to mental health resources) also buffers against burnout, but sustained improvement requires addressing systemic challenges such as staffing shortages and excessive workloads [18,19]. Stress among ICU night-shift nurses is closely linked to decreased care quality and increased patient safety risks, increasing nurses’ intention to leave, which further perpetuates the cycle of stress [5,17]. To mitigate these risks, studies recommend implementing organizational strategies, such as improved scheduling, fostering teamwork, and ensuring access to mental health support [2,5,20,21].

Despite extensive global research on ICU night-shift stress, studies from Egypt remain scarce. ICU night-shift nurses in Egypt represent a critical yet underexplored population facing context-specific challenges that affect health and job performance. This study addresses the gap by investigating work-related stress among Egyptian ICU night shift nurses, where factors such as nurse-patient ratios, staffing policies, and 12-hour shifts may shape stress differently than in other settings. Prior research also documents significant sleep disturbances in this population, which can adversely affect their quality of life and patient care [22]. Moreover, a study conducted at Ain Shams University Hospitals found that night-shift nurses are at increased risk for dyslipidemia and unhealthy lifestyle factors [23] (Gadallah, Hakim, Mohsen, & Eldin, 2017). Another study revealed that the Egyptian ICU nurses face distinct challenges, including high workloads, a lack of participation in scheduling, and significant family and social conflicts due to night shifts [24]. For that, the findings aim to identify the unique stressors these nurses encounter and to inform organizational strategies that can mitigate burnout and improve patient safety. By focusing on this underexplored setting, the study provides critical insights to strengthen nurse well-being and improve healthcare outcomes.

## Method

### Study design

A descriptive cross-sectional research design was employed in this study. This design was suitable for the research aim, as it allowed for the assessment of work-related stress and associated factors among ICU night shift nurses at a single point in time, providing a snapshot of the current state without requiring follow-up. The approach enabled efficient data collection from a relatively large sample within a limited timeframe.

### Setting

This study was conducted at the ICUs of Main Mansourah University Hospital, Convalescence Hospital, and the Internal Medicine building. These units provide direct care for all critically ill patients. Across sites, the number of ICUs ranged from three to ten, and bed capacity ranged from 14 to 40 beds. These units are well-equipped with advanced monitoring and life support technology and provide direct care to critically ill patients in general surgical, respiratory, neurosurgical, and neurological ICUs, as well as the stroke unit**.** The nurse-patient ratio in these ICUs typically ranged from 1:2–1:1, depending on the type of ICU and the patients’ conditions.

### Study sample

A convenience sample of 362 ICU nurses was recruited from the selected ICUs. Despite its limitations, this approach provided valuable insights into examining work-related stress and associated factors among ICU night shift nurses. Data were collected between January 10, 2025, and April 30, 2025. The inclusion criteria were nurses directly involved in patient care, with at least one year of experience in the ICU, and a willingness to participate in the study. Exclusion criteria included nurses in administrative roles, those on leave, and those not working night shifts. Participants who met the inclusion criteria were approached in person within their ICUs, where the study purpose and procedures were explained. Nurses who agreed to participate and signed written informed consent provided their official email addresses and subsequently received a Google Form link through which data were securely collected via email to prevent duplicate entries and ensure only eligible participants responded. This approach facilitated efficient, confidential, and contactless data collection. In accordance with the principle of voluntariness, all participants who completed the questionnaire were considered to have given their informed consent, and they were free to withdraw from the study at any time during the study period. The final sample size reflected the number of eligible and willing participants available during the study period. It exceeded the calculated sample size (= 277) using an Excel calculation sheet [25,26]:


$$n=\;\frac{{\left(Z_{\frac{\alpha }{\text{2}}}+Z_{\beta }\right)}^{\text{2}}[P(\text{1}-P)]}{d^{\text{2}}},$$

Where n is then corrected for the population size (N = 450) as $n^{*}=\frac{nN}{n+(N-\text{1})}$. Where P is the assumed population proportion (= 0.35), d is the margin of error (= 0.05), α is the significance level (= 0.05), β = 0.20 (i.e., statistical power = 80%), Zα_/2_ and Z_β_ (1.96 and 0.845, respectively) are the standard normal values.

### Data collection tool

One tool was utilized to collect data after reviewing recent relevant literature [27–29].

***Part I: Nurses’ Demographic Data Sheet:*** This section was used to gather information about nurses’ age, gender, educational level, years of work experience in the ICU, current position, working hours, and attendance at training courses on stress management.

***Part II: Revised Nursing Stress Scale (RNSS):*** This tool was adapted from Pavek et al. [27]. The Revised Nursing Stress Scale is a valid and reliable tool recommended for assessing the frequency and significant sources of stress experienced by nurses. The RNSS consisted of a total of 66 items and nine subscales as follows: death and dying, conflict with providers, inadequate preparation, problems with peers, problems with supervisors, workload, uncertainty concerning treatment, patients and their families, and discrimination. Each item was measured using a unipolar 4-point Likert scale to measure the frequency of occupational stressors as experienced by the participant. Likert scale responses consisted of never (0), occasionally (1), frequently (2), and very frequently (3). Total scores ranged from 0 to 198, with higher total scores representing higher levels of perceived. Total RNSS scores were first transformed into Sten scores (scale from 1 to 10). Sten scores were then classified into low (1–4), moderate (5–6), and high scores (7–10) [2,30]. The RNSS demonstrated acceptable to excellent internal consistency reliability (αs ≥ 0.73 and ω ≥ 0.80). This tool was translated into Arabic using a forward-backward translation technique to ensure cultural equivalence. Permission to use the RNSS was obtained from the author.

### Validity and reliability of the tool

The RNSS was translated using a standard forward-backward translation technique to ensure linguistic accuracy and cultural relevance. Two independent bilingual translators first translated the original English version into Arabic (forward translation). Another set of translators, blinded to the original, then back-translated the Arabic version into English. The back-translated version was compared with the original to identify discrepancies. An expert panel of critical care nursing professionals reviewed all versions for clarity, cultural appropriateness, and contextual relevance. Revisions were made based on their feedback to improve comprehensibility and applicability in the local nursing context. This rigorous process ensured semantic, idiomatic, experiential, and conceptual equivalence between the original and translated instruments.

The reliability analysis shows that all subscales of the RNSS have acceptable to excellent internal consistency, with Cronbach’s alpha values ranging from 0.708 to 0.942. The overall RNSS scale has a very high reliability (α = 0.967), indicating that the instrument is highly consistent in measuring work-related stress among ICU nurses. These support the use of the RNSS in this population.

### Pilot study

Before starting the data collection, a pilot study was conducted with 36 nurses, representing 10% of the overall sample, to test the feasibility, applicability, and simplicity of the translated tool. The participants who were involved in the pilot study were excluded from the main study. Participants provided feedback on the clarity of language, response options, and the relevance of items to their work environment. Minor adjustments were made based on pilot findings to enhance the tool’s usability without changing the core content or structure.

### Ethical considerations

Ethical approval for this study was obtained from the Research Ethics Committee of the Faculty of Nursing, Mansoura University (**Ref. No. 0696)**. The study was conducted in accordance with the ethical principles outlined in the Declaration of Helsinki. Informed consent was obtained from the study nurses after they were informed of the study’s aim and nature. Participants were assured of their voluntary participation and the right to withdraw from the research at any time without facing any consequences. They were also informed that their personal information was encrypted and kept confidential, and that the observed practice would not be taken into account in their annual appraisal.

### Data collection process

The researcher collected data from January to June 2025. Before data collection, the researchers introduced themselves to the participating nurses in each unit, described the purpose and nature of the study, and invited them to participate in the study. Official email addresses were obtained from participants who agreed to participate. After securing all necessary approvals, the study questionnaire was converted into a user-friendly Google Form. The link to the form was then sent to participants via their official email addresses. This secure online platform facilitated efficient and confidential data collection.

### Statistical analysis

The data were analyzed using the Statistical Package for Social Sciences (SPSS), version 21.0. Chronbach’s α was obtained as a measure of reliability. Descriptive statistics (frequencies, percentages, means, and standard deviations) were used to summarize the data. For age and education categories with very low frequency, pooling was performed with other categories, resulting in two categories for each (Age: < 30 years, ≥ 30 years; Education: less than bachelor’s, bachelor’s or higher). The distribution of stress scores (total RNSS scores and the subscale scores) was assessed graphically using boxplots and standard probability plots. Where the normality assumption was met (death and dying, problems with supervisors, patients, and their families ‘ workload, and discrimination), an independent samples t-test was used to test differences in mean scores among levels of independent variables with two categories (age, gender, having children, and education). In comparison, one-way ANOVA was carried out to test differences among levels of variables with three or more categories (marital status, work experience, and number of night shifts per week). For dependent variables where significant deviations from normality were detected (conflict with providers, inadequate preparation, problems with peers, and uncertainty concerning treatment subscales), the non-parametric Mann-Whitney U test and Kruskal-Wallis test were used instead of the t-test and One-Way ANOVA, respectively. Multiple pairwise comparisons were performed using Tukey’s test for One-way ANOVA and Dunn’s procedure with Bonferroni adjustment for the Kruskal-Wallis test.

## Results

As shown in Table 1, the majority of participants were females (91.2%) and under 30 years of age (74.9%), suggesting a young and predominantly female ICU nursing workforce. Most nurses were married (78.5%) and had children (74%), and the most common educational level was Technical Nursing Institute (67.4%). Almost half had less than 5 years of ICU experience, which may affect their stress perception. These demographics offer valuable context for understanding stress levels.

**Table 1:** Basic Characteristics of the Study Sample.

Characteristic	N	%
**Age (Years)**		
**< 30**	271	74.9
**30-40**	87	24.0
**> 40**	4	1.1
**Gender**		
Male	32	8.8
Female	330	91.2
**Marital Status**		
Single	69	19.1
Married	284	78.5
Divorced or a widow	9	2.5
**Children**		
Yes	268	74.0
No	94	26.0
**Educational Level**		
Secondary nursing school	2	0.6
Technical Nursing Institute	244	67.4
BSc Nurse	115	31.8
Postgraduate study	1	.3
**ICU Work Experience (Years)**		
< 5	180	49.7
5-10	133	36.7
> 10	40	11.0
Missing	9	2.5
**No. of Night Shifts (Week)**		
**3**	249	68.8
4	39	10.8
≥ 5	74	20.4

Table 2 presents the categorical distribution of stress levels based on the RNSS Scale. Approximately 22.1% of nurses experienced high stress, and 37.6% moderate stress, indicating that nearly 60% of the sample suffer from at least moderate levels of stress. The categorical distribution supports the continuous RNSS scores and should be considered in any targeted intervention planning.

**Table 2:** Stress Levels Based on the RNSS Scale.

Stress Category	Frequency	Percent	Valid Percent	Cumulative Percent
Low stress	146	40.3	40.3	40.3
Moderate stress	136	37.6	37.6	77.9
High stress	80	22.1	22.1	100.0
**Total**	362	100.0	100.0	

Stress scores based on the RNSS scale domain are shown in Table 3. Workload had the highest mean stress score (21.11 ± 11.12), followed by “Patients and their families” (14.84 ± 7.83) and “Problems with supervisors” (11.64 ± 7.82). This suggests that task-related and interpersonal sources of stress are the most dominant. The lowest mean score was for “Discrimination” (2.80 ± 2.73). These results highlight the critical areas that require intervention to reduce stress among ICU nurses.

**Table 3:** Mean Stress Score Across RNSS Domains Among ICU Nurses.

Stress Scale	No of items	Min	Max	Mean	Std. Deviation
Death and dying	6	0	18.0	8.49	4.00
Conflict with providers	4	0	12.0	3.35	2.60
Inadequate preparation	8	0	24.0	6.83	4.11
Problems with peers	4	0	12.0	3.31	2.75
Problems with supervisors	10	0	30.0	11.64	7.82
Work load	15	0	45.0	21.11	11.12
Uncertainty concerning treatment	6	0	18.0	4.73	3.76
Patients and their families	10	0	30.0	14.84	7.83
Discrimination	3	0	9.0	2.80	2.73
**Overall RNSS Score**	**66**	**0**	**174.0**	**77.10**	**35.89**

### Relationship of work stress with socio-demographic and work-related factors

No significant relationship (P > 0.05) was found between work stress and any of the following variables: age, marital status, education, having children, or the number of weekly night shifts. A significant relationship (P < 0.05) was found with gender concerning death and dying, patients and their families, and discrimination, where females had higher average stress scores in all three domains (Table 4).

**Table 4:** Work Stress by Gender.

Stress Scale	Males		Females		P value ^1^	95% Confidence intervals ^2^
	Mean	SD	Mean	SD		
Death and dying	7.13	3.89	8.62	3.99	**0.043**	**(0.043, 2.94)**
Conflict with providers	3.38	2.71	3.35	2.60	0.951	
Inadequate preparation	6.22	4.82	6.88	4.04	0.239	
Problems with peers	3.28	2.94	3.32	2.74	0.892	
Problems with supervisors	11.47	8.84	11.65	7.73	0.900	
Work load	19.25	10.85	21.29	11.15	0.322	
Uncertainty concerning treatment	4.13	3.59	4.79	3.77	0.362	
Patients and their families	11.59	7.67	15.15	7.79	0.014	(0.73, 6.39)
Discrimination	1.88	2.51	2.89	2.74	0.045	(0.025, 2.01)
**Overall RNSS Score**	**68.31**	**37.98**	**77.95**	**35.62**	**0.147**	

1, 2Independent samples t-test for Death and dying, Problems with supervisors, Patients and their families, Workload, and discrimination. Mann-Whitney U test for Conflict with providers, Inadequate preparation, Problems with peers, and Uncertainty concerning treatment. Confidence intervals are reported only for significant tests.

For marital status, a significant relationship was found only with the “inadequate preparation” subscale (P = 0.041), where single nurses had a higher average score (7.841) than married (6.59) and divorced or widowed nurses (6.44). A significant relationship (P = 0.023) was also found between work experience and “inadequate preparation”. Nurses with less than 5 years of experience had higher average stress scores in this subscale than nurses with 5–10 years of experience and nurses with more than 10 years of experience, as shown in Table 5. No significant relationship (P > 0.05) was found between work experience and the other stress scales or the overall RNSS score. However, the relationship was substantial (P = 0.057) with “uncertainty concerning treatment,” where nurses with less than 5 years of experience had higher average scores than those with more years of experience, as presented in Table 5.

**Table 5:** Work stress from ICU work experience.

Stress Scale	ICU Work Experience (Years)						P value ^1^
	< 5 years (N = 180)		5 −10 years (N = 133)		> 10 years (N = 40)		
	Mean	SD	Mean	SD	Mean	SD	
Death and dying	8.54	3.92	8.66	4.12	8.08	4.16	0.721
Conflict with providers	3.41	2.49	3.38	2.78	3.28	2.69	0.772
Inadequate preparation	**7.27** ^**a, 2**^	**3.80**	**6.44** ^**b**^	**4.49**	**6.35** ^**a,b**^	**4.26**	**0.023**
Problems with peers	3.56	2.76	2.93	2.68	3.40	2.98	0.090
Problems with supervisors	11.49	7.96	11.68	7.50	12.38	8.57	0.814
Work load	21.87	11.42	19.79	10.55	22.15	12.06	0.221
Uncertainty concerning treatment	5.19	4.04	4.09	3.25	4.63	3.71	0.057
Patients and their families	14.63	7.65	15.28	7.93	13.60	8.13	0.469
Discrimination	2.93	2.83	2.62	2.55	2.73	2.88	0.780
**Overall RNSS Score**	78.88	36.45	74.86	34.76	76.58	39.00	0.766

^1^ Based on One-Way ANOVA for Death and dying, Problems with supervisors, Patients and their families, Workload and discrimination, and Kruskal-Wallis test for Conflict with providers, Inadequate preparation, Problems with peers, and Uncertainty concerning treatment subscales.

^2^ Different superscripts within the same row indicate a significant difference (P < 0.05) based on Tukey’s test for One-way ANOVA and Dunn’s procedure with Bonferroni adjustment for the Kruskal-Wallis test. Although actual means are reported, the Kruskal-Wallis test is based on mean ranks, not actual means.

## Discussion

The Revised Nursing Stress Scale (RNSS) used in this study demonstrated strong internal consistency across its subscales, confirming its reliability in measuring work-related stress among ICU nurses. This aligns with a growing body of literature emphasizing the importance of validated, robust instruments for monitoring nurse stress and burnout effectively in the planning of interventions [31]. Recent research has highlighted the importance of accurately assessing stress in developing resilience-building strategies and Emotional intelligence training, particularly in high-intensity settings such as ICUs [32,33]. Ghanei Gheshlagh et al. [31] supported the reliability of the scale among Persian-speaking emergency nurses. Despite its strengths, concerns about cultural applicability remain. Nonetheless, the RNSS proved effective in the current study population, reinforcing its utility in diverse ICU environments.

The demographic profile of the sample, which is predominantly comprised of young female nurses under 30 with limited ICU experience, reflects broader regional workforce trends. High turnover and burnout among younger nurses have been documented across the Middle East and Asia [34,35]. Early-career nurses often face elevated stress due to limited coping mechanisms and clinical exposure [36]. However, age alone may not predict stress levels. Studies have shown that older nurses also experience significant stress and burnout, particularly in high-pressure ICU environments. For instance, Mathew et al. [37] found that nurses with more than five years of ICU experience still reported high levels of stress and burnout, mainly due to systemic issues such as inadequate staffing and logistical challenges.

Similarly, Villagracia et al. [38] reported that burnout among ICU nurses was prevalent across all age groups, with older nurses experiencing higher levels of emotional exhaustion and a reduced quality of life. Beier et al. [39] also highlighted that while older nurses may employ more positive coping strategies, they are not immune to burnout, especially during crises such as the COVID-19 pandemic. The finding that nearly 60% of ICU nurses reported moderate to high stress levels is consistent with studies from Nepal, Saudi Arabia, and Palestine [2,5,40,41]. This highlights the importance of effective interventions at the organizational level, such as stress management programs and supportive policies, which are necessary to mitigate stressors in the ICU environment. In contrast, Armstrong et al. [42] observed lower stress levels in Australian ICUs, attributing this to better staffing ratios and institutional support, highlighting the influence of systemic factors on stress outcomes.

Workload emerged as the most significant stressor, followed by patient-family interactions and supervisory challenges. These findings are consistent with Seok et al. [43], who identified workload and interpersonal tensions as primary stressors in ICU settings. Mathew et al. [37] found that ICU nurses frequently experience high stress due to work overload, inadequate staffing, and lack of managerial support, which directly impacts patient safety. Lima et al. [44] confirmed that burnout in ICU nurses is often driven by prolonged work periods and insufficient resources, especially during the COVID-19 pandemic. Ethical dilemmas also play a critical role in emotional exhaustion. Kalaycioglu et al. [45]demonstrated that ICU nurses facing ethical conflicts during triage and resource allocation reported significantly higher levels of emotional exhaustion and turnover intention. Similarly, Afenigus and Sinshaw [46] highlighted that moral distress from ethical decision-making in critical care settings contributes to burnout and ethical fatigue

Gender differences were evident in domains such as death and dying, patient interactions, and discrimination, with female nurses reporting higher stress levels. Jarrad et al. [47] found that female nurses experienced elevated compassion fatigue and depression, although resilience scores were similar across genders. Yalçın et al. [48] emphasized that emotional labor behaviors, particularly surface acting, were more prevalent among female nurses and significantly associated with burnout. This suggests that female nurses experience higher levels of stress compared to their male counterparts, influenced by various factors including workload, dual role conflicts, and workplace dynamics [2]. Additionally, the dual role conflict, where female nurses juggle professional responsibilities and domestic duties, correlates strongly with increased stress [2,49]. These findings suggest that gender-sensitive interventions and emotional support strategies may be beneficial in mitigating stress among ICU nurses.

Experience in ICU settings was significantly associated with stress related to treatment uncertainty. Less experienced nurses reported higher stress, likely due to unfamiliarity with protocols and lower clinical confidence. Moreover, research indicates that structured support systems, such as mentorship programs, are crucial for easing the transition from education to practice, thereby reducing stress and enhancing confidence in clinical skills [50]. Mathew et al. [37] found that ICU nurses with fewer years of experience were more vulnerable to stress and burnout, which negatively impacted patient safety. Similarly, Liu et al. [51] emphasized that psychological resilience among ICU nurses is influenced by training, workload, and organizational support, with less experienced nurses requiring more targeted interventions. These findings support the notion that experience fosters cognitive resilience and decision-making capacity in critical care. However, prolonged exposure to ICU environments may also lead to cumulative stress and burnout. Villagracia et al. [38] reported that ICU nurses with longer tenure still experienced high levels of burnout, which correlated negatively with their quality of life. This suggests that while experience may buffer specific stressors, it does not universally protect against the psychological demands of ICU work.

Organizational interventions have shown promise in mitigating stress and improving nurse well-being. Furthermore, reducing stress among ICU nurses is significantly influenced by organizational and structural contributors, particularly staffing shortages and leadership dynamics. Addressing these factors is crucial for enhancing the work environment and overall nurse well-being [2,5,52]. Xavier et al. [53] emphasized the importance of resilience-building strategies tailored to the needs of ICU nurses. Mealer et al. [54] demonstrated the feasibility and acceptability of a multimodal resilience training program, including mindfulness and cognitive behavioral techniques, which reduced PTSD symptoms among ICU nurses.

### Limitations

The study has several limitations. First, its cross-sectional design limits the ability to establish causal relationships between variables. Future research is warranted to include intervention-based studies and longitudinal designs. Furthermore, the use of a convenience sample from a single hospital network limits the generalizability of the findings to other settings. Second, data were collected using a self-report questionnaire, which may be subject to response bias.

Third, the absence of qualitative data limited deeper exploration of nurses’ lived experiences and perceptions. Additionally, essential variables such as organizational support, workplace culture, burnout, job performance, leadership style, and coping strategies were not addressed, although they may also influence stress levels. Future research is recommended to incorporate longitudinal and mixed-methods approaches, include these additional variables, and expand the scope across multiple hospitals and regions to enhance the applicability and depth of the findings.

**Conclusion:** Work-related stress is highly prevalent among ICU nurses working night shifts, primarily driven by heavy workloads and interpersonal challenges. Stress levels vary significantly by gender and years of experience, underscoring the need for organizational interventions, such as staff support programs, training, effective shift management, flexible scheduling, and psychosocial support, to mitigate stress and improve the quality of care.

### Recommendations

Based on the current findings, the following are recommended. Healthcare organizations should implement targeted stress management programs for ICU night shift nurses to reduce work-related stress and its negative impact on well-being and patient care quality. Strategies such as cognitive-behavioral training, mindfulness, peer support groups, and workload adjustments can help nurses enhance their coping skills and resilience. Nursing education programs should incorporate training on recognizing stress, developing effective coping mechanisms, and practicing self-care strategies to promote overall well-being. Ongoing professional development and workshops focusing on mental health and stress reduction are crucial for preparing nurses for the high demands of ICU environments. Moreover, further studies should adopt longitudinal designs to explore the progression of work-related stress and evaluate the effectiveness of intervention programs over time. Research expanding to multiple hospitals and diverse healthcare settings will improve the generalizability of findings. Additionally, investigating organizational culture, leadership styles, and individual coping strategies can provide a holistic understanding of factors influencing nurse stress and burnout.

## Supporting information

S1 File S1–S6 Tables provide extended Basic Characteristics of the Study Sample, Stress Levels Based on the RNSS Scale, detailed statistical outputs across RNSS Domains Among ICU Nurses, and approval documentation. (DOCX)

S1 Data Work stress Excel data 2025. (XLSX)

## Abbreviations

RNSS: revised nursing stress scale
ICU: intensive care unit.
