# Supplementary material for: Work-related stress in intensive care unit night shift nurses: A cross-sectional analysis of prevalence and determinants
Source: PLoS One. 2025 Nov 10;20(11):e0336041. doi: 10.1371/journal.pone.0336041 (PMC12599929; doi:10.1371/journal.pone.0336041)
Supplement: S1 File — (DOCX) [file pone.0336041.s001.docx]

**Supporting Information**

S1 Table. Basic Characteristics of the Study Sample

• Age
 – < 30 years: 271 (74.4.8%)
 – 30-40 years: 87 (24.0%)
 – > 40years: 4 (1.1%)

• Gender
 – Male: 32 (8.8%)
 – Female: 330 (91.2%)

• Marital Status
 – Married: 284 (78.5%)
 – Single: 69 (19.1%)

–Divorced or a widow: 9(2.5%)

• Children

–Yes: 268(74%)

–No: 94(26.0%)

•Educational Level:

–Secondary nursing school: 2(0.6%)

– Technical Nursing Institute: 244(67.4%)

– BSc Nurse: 115(31.8%)

– Postgraduate study: 1(0.3%)

•ICU Work Experience (Years)

–< 5 : 180(49.7)

–5-10: 133(36.7)

–> 10 : 40(11.0%)

–Missing: 9(2.5%)

• No. of Night Shifts (Week)

– 3: 249( 68.8%)

– 4: 39(10.8%)

– ≥ 5:74(20.4%)

S2 Table. Stress Levels Based on the RNSS Scale
 – Low stress: 146(40.3%)
 – Moderate stress: 136(37.6%)

– High stress: 80(22.1%)

S3 Table. Mean Stress Score Across RNSS Domains Among ICU Nurses
• Overall RNSS Mean Score: 77.10 ± 35.89

S4. Table. Work Stress by Gender
• Overall RNSS Score: Male 68.31 ± 37.98, Female: 77.95± 35.62; p-value: 0.147

S5. Table. Work Stress from ICU work experience

• Overall RNSS Score: Male 68.31 ± 37.98, Female: 77.95± 35.62; p-value: 0.147

–< 5 : 78.88± 36.45, 5-10: 74.86± 34.76, > 10 : 76.58± 39.00; p-value: 0.766

–Missing: 77.95± 35.62

S6 Text. Ethical Approval Documentation

• Project Title: Work-Related Stress among Night Shift Nurses Working in Intensive Care Units
• Project Number: 0696
• Approval Date: January 9, 2025

S7 Methods. Statistical Analysis Details

• Software Used: IBM SPSS Statistics Version 21
• Analyses Conducted: Descriptive statistics, t-tests, one-way ANOVA. Multiple pairwise comparisons were performed using Tukey’s test for One-way ANOVA and Dunn’s procedure with Bonferroni adjustment for the Kruskal-Wallis test.

• Significance Threshold: P < 0.05
